# Extracellular organic disulfide reduction by *Shewanella oneidensis* MR-1

**DOI:** 10.1128/spectrum.04081-23

**Published:** 2024-02-28

**Authors:** Jonathan Phan, Shine Macwan, Jeffrey A. Gralnick, Nathan Yee

**Affiliations:** 1 School of Environmental and Biological Sciences, Rutgers University, New Brunswick, New Jersey, USA; 2 Department of Plant and Microbial Biology, BioTechnology Institute, University of Minnesota, Twin Cities, Minnesota, USA; Politecnico di Torino, Torino, Piemonte, Italy

**Keywords:** DTNB, TNB, outer membrane, organic sulfur, thiol, *Shewanella*, cysteine, cystine, glutathione, geomicrobiology

## Abstract

Microbial reduction of organic disulfides affects the macromolecular structure and chemical reactivity of natural organic matter. Currently, the enzymatic pathways that mediate disulfide bond reduction in soil and sedimentary organic matter are poorly understood. In this study, we examined the extracellular reduction of 5,5′-dithiobis(2-nitrobenzoic acid) (DTNB) by *Shewanella oneidensis* strain MR-1. A transposon mutagenesis screen performed with *S. oneidensis* resulted in the isolation of a mutant that lost ~90% of its DTNB reduction activity. Genome sequencing of the mutant strain revealed that the transposon was inserted into the *dsbD* gene, which encodes for an oxidoreductase involved in cytochrome *c* maturation. Complementation of the mutant strain with the wild-type *dsbD* partially restored DTNB reduction activity. Because DsbD catalyzes a critical step in the assembly of multi-heme *c*-type cytochromes, we further investigated the role of extracellular electron transfer cytochromes in organic disulfide reduction. The results indicated that mutants lacking proteins in the Mtr system were severely impaired in their ability to reduce DTNB. These findings provide new insights into extracellular organic disulfide reduction and the enzymatic pathways of organic sulfur redox cycling.

**IMPORTANCE:**

Organic sulfur compounds in soils and sediments are held together by disulfide bonds. This study investigates how *Shewanella oneidensis* breaks apart extracellular organic sulfur compounds. The results show that an enzyme involved in the assembly of *c*-type cytochromes as well as proteins in the Mtr respiratory pathway is needed for *S. oneidensis* to transfer electrons from the cell surface to extracellular organic disulfides. These findings have important implications for understanding how organic sulfur decomposes in terrestrial ecosystems.

## INTRODUCTION

Sulfur (S) redox cycling influences carbon respiration, nutrient availability, and contaminant sequestration in soils and sediments (1–3). Organic S is the primary form of sulfur in many terrestrial ecosystems (4, 5), and the oxidation of thiols in organic S produces disulfide bonds (R-S-S-R) which strongly affect the macromolecular structure and reactivity of natural organic matter (6, 7). The reduction of disulfide bonds in organic matter is a key environmental process that breaks up disulfide cross-linkages and liberates high-affinity sulfhydryl functional groups that are involved in trace nutrient binding and metal complexation (8, 9). Currently, the microbial processes that mediate disulfide bond reduction in soil and sedimentary organic matter are poorly understood.

*Shewanella oneidensis* strain MR-1 is a facultative anaerobe capable of catalyzing a diverse array of reduction reactions (10). This Gram-negative bacterium harbors a large number of multi-heme *c*-type cytochromes that facilitate the extracellular reduction of inorganic and organic substrates (11), including dissolved organic compounds such as flavins, quinones, and humic acids (12–14). Previous studies have shown that *S. oneidensis* can transfer electrons extracellularly to nitrogen atoms in riboflavin and riboflavin-5′-phosphate (15) and carbon atoms in the humic acid analog anthraquinone-2,6-disulfonate (16). Humic acids also contain disulfide bonds (7, 17), but very little is known about extracellular electron transfer to organic S in humic substances.

In this study, we conducted experiments to identify the genes involved in extracellular organic disulfide reduction by *S. oneidensis*. We employed transposon mutagenesis to isolate a *S. oneidensis* mutant impaired in organic disulfide reduction. The location of the transposon insertion was identified, and genetic complementation was carried out to restore the loss of disulfide reduction activity. *S. oneidensis* mutants that lacked outer-membrane cytochromes were also screened to further elucidate the mechanism of extracellular disulfide bond reduction. The results provide new insights into the enzymatic pathways that catalyze the breakup of organic disulfide bonds.

## RESULTS AND DISCUSSION

**Fig 1:**
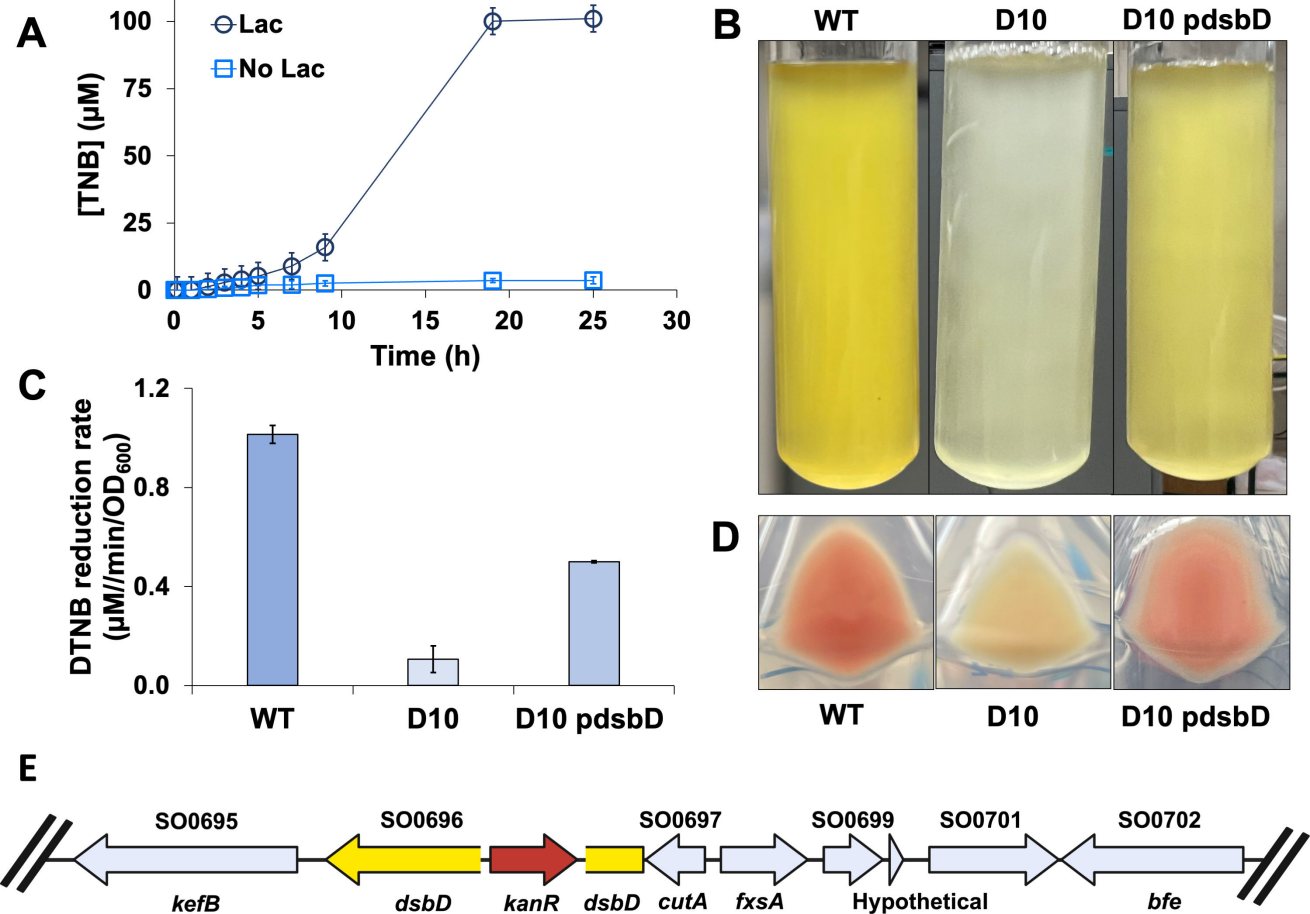
DTNB reduction by the wild-type and mutant strains. (**A**) Reduction of DTNB to TNB by the wild-type strain in M1 medium with and without lactate. Each data point represents the average of three replicate experiments, and error bars indicate the standard deviation. (**B**) Overnight cultures grown in M1 lactate medium supplemented with 100 µM DTNB; wild-type (left), D10 mutant (middle), and complemented mutant D10 pdsbD (right). (**C**) Initial velocity of DTNB reduction for the wild-type, D10, and D10 pdsbD strains. (**D**) Cell pellets centrifuged; wild-type (left), D10 (middle), and D10 pdsbD (right). (**E**) Location of transposon insertion. The *dsbD* gene in yellow and the transposon in red.

*S. oneidensis* reduced the organic disulfide 5,5′-dithiobis-(2-nitrobenzoic acid) (DTNB) in an anaerobic M1 medium with lactate to form yellow-colored 2-nitro-5-thiobenzoate (TNB) (Fig. 1A). Complete DTNB reduction was observed in less than 20 hours of incubation. Cell suspensions in the M1 medium without lactate did not reduce DTNB. After screening 9,348 transposon mutants, a single mutant, designated as strain D10, was isolated, which was impaired in DTNB reduction. D10 did not produce a visible yellow-colored product (Fig. 1B) and lost approximately 90% of its DTNB reduction activity (Fig. 1C). D10 cells also lost the pink coloration characteristic of *S. oneidensis* (Fig. 1D). Genome sequencing of D10 revealed a transposon insertion within the *dsbD* gene (SO0696) (Fig. 1E), which encodes for an oxidoreductase involved in cytochrome *c* maturation (18). Complementation of the mutation using the wild-type (WT) *dsbD* sequence partially restored the abolished phenotype (Fig. 1C and D). The complemented mutant exhibited ~65% of the DTNB activity of MR-1, and pink coloration returned to the cells.

**Fig 2:**
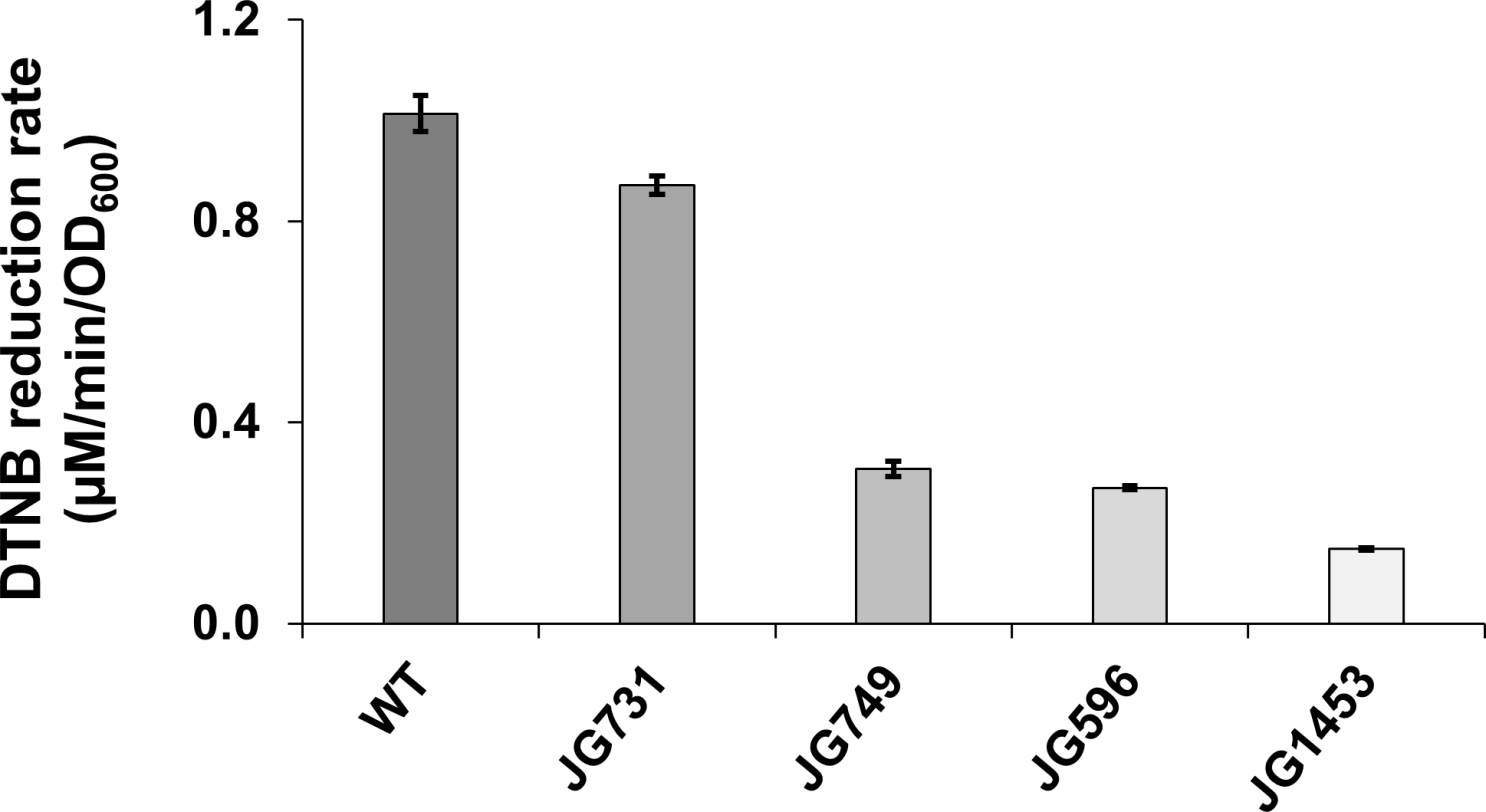
DTNB reduction by Mtr mutant strains JG731 (Δ*mtrC*), JG749 (Δ*mtrC*/Δ*omcA*), JG596 (Δ*mtrC*/Δ*omcA*/Δ*mtrF*), and JG1453 (Δ*mtrB*/Δ*mtrE*/Δ*mtrC*/Δ*omcA*/Δ*mtrF*/Δ*mtrA*/Δ*mtrD*/Δ*dmsE*/Δ*SO4360*/Δ*cctA*). Experiments were performed in triplicate, and error bars represent the standard deviation. Statistically significant differences were observed between the WT and all mutant strains (Student’s *t*-test, *P* < 0.01).

Because the *dsbD* gene is involved in cytochrome *c* maturation, we sought to investigate the role of the *S. oneidensis* Mtr system in extracellular disulfide bond reduction. Mutant strains JG731 (Δ*mtrC*), JG749 (Δ*mtrC* Δ*omcA*), JG596 (Δ*mtrC* Δ*omcA* Δ*mtrF*), and JG1453 (Δ*mtrB* Δ*mtrE* Δ*mtrC* Δ*omcA* Δ*mtrF* Δ*mtrA* Δ*mtrD* Δ*dmsE* Δ*SO4360* Δ*cctA*) were tested for DTNB reduction activity. Mutants lacking the outer-membrane decaheme c-type cytochrome MtrC reduced DTNB at approximately 90% of the wild-type strain. Mutants lacking both the decaheme outer-membrane *c*-type cytochromes MtrC and OmcA lost more than half of their DTNB reduction activity. Additional deletion of the decaheme outer-membrane cytochrome MtrF had little effect on DTNB reduction. Mutant strain JG1453, which harbors two outer-membrane beta barrel deletions (MtrB and MtrF) and eight cytochrome deletions, reduced DTNB at less than 15% of the rate of MR-1 (Fig. 2).

### Enzymatic pathway of DTNB reduction

The results indicate that the mutation of the *dsbD* gene in *S. oneidensis* impairs DTNB reduction. DsbD is an inner-membrane protein that plays a critical role in the assembly of multi-heme *c*-type cytochromes in the periplasm. When apocytochromes are secreted into the periplasm, the cysteine residues associated with the heme-binding motifs (CxxC) are immediately oxidized. For heme ligation to occur, DsbD must convert the oxidized cysteines back to the reduced state (19). This enzymatic step is carried out by DsbD transferring electrons from the cytoplasmic thioredoxin system across the inner membrane to periplasmic apocytochromes through thiol-disulfide exchange reactions (20). Loss of DsbD disables this pivotal step in cytochrome maturation resulting in the inability of *S. oneidensis* to respire certain substrates anaerobically. Previous studies have shown that *dsbD* mutants are unable to respire cobalt (21) or transfer electrons extracellularly to electrodes (22). Beyond these phenotypes, our results demonstrate that a functional DsbD enzyme in *S. oneidensis* is also needed for the extracellular reduction of DTNB.

We observed that *dsbD* mutant cells lacked the distinctive pink coloration exhibited by the wild-type strain (Fig. 1D), which suggested that the absence of red-colored cytochromes was related to the loss of DTNB reduction activity (23, 24). The data presented in Fig. 2 show that mutants with a functional DsbD oxidoreductase but deletions in the Mtr system are greatly subdued in DTNB reduction. Anaerobic cultures of the wild-type strain incubated with lactate reduced DTNB (Fig. 1A) indicating that electrons are transferred from the electron transport chain via Mtr proteins to break apart extracellular organic disulfides. The Mtr system receives electrons from periplasmic electron carriers that regulate extracellular electron transfer efficiency (25). We observed progressive loss of DTNB reduction activity with successive cytochrome gene deletions, similar to other substrates that are dependent on the Mtr pathway (26–28).

Together, these data suggest that DTNB reduction occurs on the cell surface, most likely via direct electron transfer reactions with heme groups in outer-membrane cytochromes. While almost all of the DTNB reduction activity can be attributed to electron transfer reactions with Mtr proteins, we noted that DTNB reduction was not completely abolished in the Mtr mutant strains (Fig. 2). The low levels of residual activity in the mutants indicate that mechanisms other than the Mtr pathway might be involved in DTNB reduction. Besides Mtr cytochromes, the genome of *S. oneidensis* contains genes for additional outer-membrane multi-heme proteins (e.g., SO1659 and SO2931) that could potentially reduce DTNB. Furthermore, because of its low molecular weight, DTNB could also pass through outer-membrane porins and interact with periplasmic cytochromes. In *S. oneidensis*, the periplasmic octaheme cytochromes SirA and OTR catalyze the reduction of inorganic sulfur compounds sulfite and tetrathionate, respectively (29, 30). Whether or not periplasmic cytochromes can also reduce organic sulfur compounds is currently unknown. Cytoplasmic DTNB reduction is less plausible. *Shewanella* species employ dedicated transporters to import naturally occurring sulfur compounds across the inner membrane, and it is unlikely that synthetic DTNB is transported into the cytoplasm for intracellular reduction.

### Environmental implications

Our results suggest that organic disulfides in soils and sediments can be biologically reduced by the Mtr system. Recent phylogenomic studies have shown that *mtr* genes are widespread in the *Shewanella* genus (10) and the Bacteria domain (31). If the Mtr system in these microorganisms can transfer electrons to disulfide bonds in naturally occurring organic matter, then our proposed enzymatic pathway has significant implications for organic sulfur redox cycling. Humic acids that participate in extracellular electron transfer reactions (12) are known to be heterogeneously cross-linked with disulfide bonds (17). The reduction of these disulfide bonds by the Mtr system would break apart humic acids, thus affecting the macromolecular structure and chemical reactivity. The extent to which this occurs in the environment would largely be dependent on the redox potential of the humic acid disulfide bonds. DTNB has a highly oxidized disulfide bond that is likely much more reactive than many naturally occurring organic disulfides. In complex molecules such as proteins, the redox potentials that have been measured for disulfide bonds range from −95 to −470 mV (32). These variations in redox potential are attributed to several factors, including sulfhydryl group pK_a_, entropy, and bond-strain energy. Similarly, humic acids are expected to harbor a large range of disulfide redox potentials, with the highest redox potential disulfide bonds being the most thermodynamically favorable for enzymatic reduction by the Mtr pathway.

Finally, the reduction of organic disulfides to thiols by *S. oneidensis* might also play a role in extracellular electron shuttling to Fe(III) minerals (33). Extracellular thiols can chemically reduce Fe(III) minerals such as ferrihydrite and smectite clays (34–37). Cysteine/cystine redox recycling by *S. oneidensis* has been shown to facilitate the reduction of ferruginous smectites (38). Cysteine-mediated Fe(III) reduction is thought to be driven by the production of reactive thiols by microbial cystine reduction, whereby extracellular cysteine acts as a conduit for electron transfer to mineral surfaces. The enzymatic reduction of naturally occurring disulfides and the coupled biotic–abiotic thiol/disulfide electron shuttling to Fe(III) minerals in soils and sediments merit further investigation.

## MATERIALS AND METHODS

### Bacterial strains and growth conditions

All strains and plasmids used in this study are listed in Table 1. Mutant strains containing gene deletions in the Mtr system were previously constructed by Coursolle and Gralnick (39). *S. oneidensis* MR-1 and its derivatives were maintained on a Luria–Bertani (LB) medium with the appropriate antibiotics. When required, the antibiotic concentrations were kanamycin at 50 µg/mL and chloramphenicol at 20 µg/mL. Experiments were conducted with cultures in an M1 lactate medium adapted from Myers and Nealson (40). This medium contained (per liter) 1.19 g (NH_4_)_2_SO_4_, 0.99 g K_2_HPO_4_, 0.45 g KH_2_PO4, 0.25 g MgSO_4_·7H_2_O, 0.07 g CaCl_2_·2H_2_O, 0.17 g NaHCO_3_, 10 mL of a trace metal solution (100×), 1 mL of a sodium selenite solution (6 mM), 14.8 mL of a sodium lactate solution (30% vol/vol), and 20 mL of an amino acid solution. The trace metal solution (100×) contained (per liter) 2.5 g Na_2_EDTA, 0.35 g H_3_BO_3_, 0.06 g NaCl, 0.15 g FeSO_4_·7 H_2_O, 0.12 g CoCl_2_·6 H_2_O, 0.13 g NiSO_4_·6 H_2_O, 0.08 g Na_2_MoO_4_, 0.02 g MnSO_4_·7H_2_O, 0.03 g ZnSO_4_·7 H_2_O, and 0.005 g CuSO_4_·7H_2_O. The amino acid solution contained (per 100 mL) 0.2 g each of serine, arginine, and glutamate.

**TABLE 1:** Strains, plasmids, and primers

Strains, plasmids, or primers	Characteristics		References
*S. oneidensis* strains			
JG274	*Shewanella oneidensis* MR1; wild type		(40)
D10	Tn insertion in *dsbD*; *kmR*; *dsbD*::[oriR6K *KmR*] (SO0696); KmR		This study
D10 pdsbD	D10 with pdsbD; KmR; CmR		This study
JG596	Δ*mtrC*/Δ*omcA*/Δ*mtrF*		(28)
JG731	Δ*mtrC*		(28)
JG749	Δ*mtrC*/Δ*omcA*		(28)
JG1453	Δ*mtrB*/Δ*mtrE*/Δ*mtrC*/Δ*omcA*/Δ*mtrF*/Δ*mtrA*/Δ*mtrD*/Δ*dmsE*/Δ*SO4360*/Δ*cctA*		(39)
*Escherichia coli* strains			
UQ950	*E. coli* host for cloning		(41)
UQ950 pdsbD	*E. coli* cloning host with pdsbD, CmR		This study
WM3064	Dap auxotroph; donor strain for conjugation		(41)
WM3064 pdsbD	*E. coli d*onor strain with pdsbD, CmR		This study
Plasmids			
pBBr-MCS1	4.7 kb broad-host range cloning vector plasmid: *CmR*, *lacZ*; 4,707 bp		(42)
pdsbD	pBBr-MCS1 with *dsbD* and 21 bp upstream and 19 bp downstream; 6,580 bp (1873 *dsbD* PCR product)		This study
Primers			
*dsbD*-F	5′-gtgagcgcgcgtaatacgactattggcgattaaactgcg; 19 bp downstream *dsbD* and includes 21 bp of T7 promoter pBBr-MCS1		This study
*dsbD*-R	5′-ctccaattcgccctatagtggataaaagataacactcagc; 21 bp upstream *dsbD* start codon and includes 20 bp of T7 promoter pBBr-MCS1		This study
pBBr-MCS1-F	5′-agtcgtattacgcgcgctcac; inside T7 promoter		This study
pBBr-MCS1-R	5′-cactatagggcgaattggag; inside T7 promoter		This study

### DTNB reduction experiment

Organic disulfide reduction experiments were performed using the cell-impermeable compound DTNB, which is a synthetic colorogenic chemical also known as Ellman’s reagent (43). The reduction of the disulfide bond in DTNB produces TNB, which is a yellow-colored product that can be quantified spectrophotometrically. DTNB reduction experiments were carried out with *S. oneidensis* MR-1 and its derivatives grown in either LB broth or M1 medium supplemented with appropriate antibiotics. After cultures were incubated aerobically at 30°C for 20 hours, the cells were harvested by centrifugation at 8,000 × *g* for 10 minutes, washed once with 0.1 M NaCl, and resuspended with deoxygenated M1 lactate medium in N_2_-purged serum bottles sealed with rubber stoppers. DTNB was then added to the cell suspensions (OD_600_ = 0.3) at a final concentration of 100 µM and incubated anaerobically at 30°C. The cell suspensions were periodically sampled to monitor cell densities (OD_600_) and the formation of TNB. The concentrations of TNB were determined by centrifuging the samples and analyzing the supernatants spectrophotometrically at 412 nm (44). TNB calibration curves were constructed by reacting DTNB with known amounts of L-cysteine. All DTNB reduction experiments were conducted in triplicate with results reported as the mean and standard deviation of the triplicate experiments. Control experiments were performed with an M1 medium without lactate. Student’s *t*-test was used to determine if differences between the wild-type and mutant strains were statistically significant.

### Transposon mutagenesis

*S. oneidensis* transposon mutants were generated previously by Brutinel and Gralnick (45). The transposon mutant library was cultured in LB broth with 50 µg/mL kanamycin, then plated on M1 lactate agar. Single colonies were picked and transferred to 96-well plates containing 250 µL of M1 lactate medium with 100 µM DTNB. Plates were then sealed in a plastic bag and incubated at 30°C. After 24 hours, cell densities and TNB concentrations were measured using a BioTek microplate reader at 600 and 412 nm, respectively. Nine thousand three hundred forty-eight transposon mutants were screened. Although genome coverage was not fully saturated, mutants that produced less than 70% of the TNB compared to the wild-type strain were isolated. These mutants were selected for further investigation.

After isolating a Tn mutant deficient in DTNB reduction, genomic DNA from the mutant strain was isolated using a Wizard HMW DNA extraction kit, and whole genome shotgun sequencing was performed by SeqCenter (Pittsburg, PA). Illumina reads were quality-checked with fastQC (46). Reads were assembled into contigs with SPAdes (47). Contigs were annotated using RAST with transposon components *oriT* and *kanR* genes used to locate the transposable element (48).

### Complementation

The wild-type *dsbD* gene was PCR-amplified with primer set *dsbD*-F and *dsbD*-R (Table 1). The PCR product was cloned into a linearized pBBr-MCS1 plasmid using the Gibson assembly method and replicated in *E. coli* UQ950. The plasmid, designated as pdsbD, was then isolated and transferred into *E. coli* WM3064. Finally, the plasmid was transferred from *E. coli* WM3064 to the *S. oneidensis dsbD* mutant by conjugal mating, and transconjugants were selected using the appropriate antibiotics. The complemented mutant was designated as D10 pdsbD.
